# Noninvasive prenatal tests for chromosomal abnormality screening in in vitro fertilisation elderly pregnant women in northwest China

**DOI:** 10.5937/jomb0-57295

**Published:** 2025-11-05

**Authors:** Shuyuan Xue, Lixia Wang, Zhen Yu, Jingying Zhu, Le Feng, Guifeng Din, Penggao Dai

**Affiliations:** 1 Northwest University, The College of Life Sciences, Xi'an, China; 2 Urumqi Maternal and Child Health Care Hospital, Prenatal Diagnosis Center, Urumqi, China; 3 Urumqi Maternal and Child Health Care Hospital, The Department of Obstetrics and Gynaecology, Urumqi, China

**Keywords:** assisted reproductive technology, in vitro fertilisation, chromosomal aneuploidy, noninvasive prenatal test, asistirane reproduktivne tehnologije, vantelesna oplodnja, hromozomske aneuploidije, neinvazivni prenatalni test

## Abstract

**Background:**

The study aimed to explore the value of using noninvasive prenatal tests (NIPT) in the second trimester of pregnancy for chromosomal abnormality screening in vitro fertilisation (IVF) in elderly pregnant women and to analyse the reasons for inconsistent screening results in northwest China.

**Methods:**

A total of 47,286 pregnant women aged 19-51 who underwent prenatal examinations were collected. NIPT detection found that the positive rate of pregnant women aged &gt; 35 with spontaneous pregnancy was 0.78% , and the positive rate of IVF pregnancy was 0.82%. Then, the detection accuracy of NIPT for pregnant women aged &gt;35 with IVF was further analysed.

**Results:**

NIPT's sensitivity, specificity, and positive predictive value in detecting common chromosomal aneuploidies (T21, T18, and T13) in pregnant women aged &gt;35 who received IVF were 99.72% , 99.78% , and 66.45% , respectively. The mean gestational age, pregnancy number, AFP, and free b-HCG significantly differed between the positive and false positive groups (P &lt; 0.05). Logistic analysis showed that the mean gestational age and AFP were independent risk factors for the failure of NIPT diagnosis (P &lt; 0.05).

**Conclusions:**

NIPT has a particular detection performance for common chromosomal aneuploidies IVF in pregnant women. However, factors affecting detection accuracies must be considered when using it.

## Abstract (sr)

**Uvod:**

Cilj istraživanja bio je da se ispita vrednost primene neinvazivnih prenatalnih testova (NIPT) u drugom trimestru trudnoce za skrining hromozomskih abnormalnosti kod trudnica starije životne dobi koje su zatrudnele putem vantelesne oplodnje (IVF), kao i da se analiziraju razlozi za neusaglašene rezultate skrininga u severozapadnoj Kini.

**Metode:**

Obuhvaceno je ukupno 47.286 trudnica uzrasta od 19 do 51 godine koje su prošle prenatalne preglede. NIPT detekcija je pokazala da je stopa pozitivnih nalaza kod trudnica starijih od 35 godina sa spontano začetom trudnocom iznosila 0,78% , dok je kod trudnoca ostvarenih putem IVF-a bila 0,82%. Zatim je dalje analizirana tačnost detekcije NIPT testa kod trudnica starijih od 35 godina koje su zatrudnele putem IVF-a.

## Introduction

Fertilisation failure in assisted reproductive technology is often unpredictable because it only occurs after in vitro fertilisation (IVF) or intracytoplasmic sperm injection [1] [2] [3] [4] [5]. The high incidence of chromosomal aneuploidy (abnormal number of chromosomes) in human gametes and embryos is a significant cause of IVF failure, although chromosomal abnormalities are about 1/150 [6] [7] [8] [9] [10]. Down syndrome (trisomy 21), Edwards syndrome (trisomy 18), and Patau syndrome (trisomy 13) are the three most common chromosomal aneuploidies [11] [12] [13] [14] [15]. Therefore, prenatal examinations during pregnancy are essential for IVF mothers to avoid unexpected events. For a long time, prenatal testing was mainly based on the biochemical indicators of the pregnant woman's blood combined with ultrasound to assess the development of the embryos [16]. Research on early pregnancy serum testing of IVF mothers found that IVF mothers have higher beta-human chorionic gonadotrophin (β-Hcg) and alpha-fetoprotein (AFP) expression levels than spontaneous pregnancy [17] [18] [19] [20]. However, some studies have shown that screening IVF mothers for serum factors may result in more false positives when chromosomal abnormalities occur in embryos, leading to an increased frequency of invasive prenatal tests (IPT) and causing unnecessary damage to the mother's body [21] [22].

With the continuous progress of clinical prenatal screening technology research, the options for genetic testing and screening in early pregnancy are increasing [23] [24]. Screening tests can determine whether an individual has an increased risk of pregnancy due to specific aneuploidies. In contrast, diagnostic tests determine whether there are particular conditions or aneuploidies in the embryo or fetus [25] [26]. Chorionic villus sampling (CVS) and amniocentesis are diagnostic methods for fetal aneuploidy and are considered the gold standard for diagnosis [27]. Historically, the main limitations of these technologies were related to their invasiveness and the possibility of pregnancy loss associated with surgery, although the surgery-related risk is less than 1% [28]. Therefore, the scientific community has been trying to identify noninvasive screening tests over the past 50 years to select women at increased risk of fetal aneuploidy to limit invasive tests' use [29]. Early pregnancy screening tests include sequential screening (maternal serum biochemical testing and fetal ultrasound markers related to maternal age) and noninvasive prenatal tests (NIPT), which use cell-free DNA (cfDNA) from the fetus [30]. NIPT is the latest innovation in prenatal diagnosis, aimed at helping clinicians diagnose pregnancy, provide counselling to pregnant women, and allow future parents to make informed choices about their unborn children [31]. Currently, the main applications for NIPT are still advanced maternal age, previous children with chromosomal abnormalities, fetal abnormalities detected by ultrasound, and a history of genetic and physical abnormalities in parents or family members [32]. Advanced maternal age increases the risk of aneuploidy in oocytes/embryos or offspring [33]. However, there is no related research on the potential interference factors that may cause false positives in using NIPT to screen for chromosomal abnormalities in IVF fetuses. This study compared the values of NIPT and IPT in screening fetal chromosomal abnormalities and found no significant difference in positive rates between the two methods. Further, the research results show that there is no significant difference in the positive rate of chromosomal abnormalities between IVF and spontaneous pregnancy in northwest China.

## Materials and methods

### Study design, setting, and ethics approval

This study was conducted at the Maternal and Child Health Hospital of Urumqi City in China. The study is a single-centre retrospective study that follows the principles of the Helsinki Declaration. Written informed consent was obtained from all patients. The hospital ethics committee of the Maternal and Child Health Hospital of Urumqi City approved the study design and any potential ethical issues (Approval number: XJFYLL2023049).

### Participants

The study included 47,286 pregnant women who visited Urumqi Maternal and Child Health Care Hospital from January 2018 to June 2022. Pregnant women are 19-51 years old, and their gestation weeks are 12-26 weeks. The prenatal screening information is provided to all pregnant women. Pregnant women can voluntarily choose Down's serological screening or NIPT screening under the premise of fully understanding the risks of prenatal screening and diagnostic techniques. The exclusion criteria for pregnant women participating in NIPT test and screening follow the Technical Specifications for Prenatal Screening and Diagnosis of Free DNA of Pregnant Women in Peripheral Blood Fetus issued by China Health and Family Planning Commission, that is, the pregnancy of the tested person is less than 12 weeks; clinical symptoms of chromosomal abnormality found in one parent; subjects had received transplantation or allogeneic cell therapy, received allogeneic blood transfusion within 1 year after pregnancy, or received cellular immunotherapy with the introduction of foreign DNA within 4 weeks after pregnancy; fetal ultrasonography indicated structural abnormalities requiring prenatal diagnosis; a family history of Down syndrome or chromosomal abnormalities that suggest a high risk of genetic disease in the fetus; the subject was pregnant with malignant tumor; the client is pregnant with three or more multiple births; other conditions that the physician considers to have a significant impact on the accuracy of the results. All pregnant women received genetic counselling and signed informed consent.

### Serological indicator testing method

3 mL of venous blood was extracted from pregnant women and centrifuged to separate the serum for testing. Enzyme-linked immunosorbent assay (ELISA) was used to screen the serum for the free beta subunit of chorionic gonadotropin (free β-hCG) using a kit purchased from Biyun Tian Biological Technology Co., Ltd. (catalogue number: AG103), and for serum alpha-fetoprotein (AFP) using a kit purchased from Shanghai Enzyme-linked Biotechnology Co., Ltd. (catalogue number: ml092666). All operations were strictly conducted following the instructions of the test kits.

### NIPT Testing Method

Ten millilitres of peripheral blood from pregnant women included in the study were collected, plasma was separated by centrifugation, and DNA was extracted by Plasma Cell-Free DNA Isolation Kit (Berry Genomics Corporation, China). A library was constructed according to the method of previous NIPT kits for fetal chromosomal aneuploidy (Berry Genomics Corporation, China). Then, the fetal chromosomal aneuploidy was detected using the high-throughput sequencer NextSeq CN500 sequencers (Illumina, San Diego, CA), and information analysis was performed using second-generation sequencing technology (next-generation sequencing, NGS) to calculate the risk values (Z values) for various chromosome abnormalities. A risk scoring method was used to determine Z 3 as high risk, -3<Z<3 as low risk, and formal reports were issued for the risk assessment results of trisomy 21, 18, and 13. Samples that failed the quality control criteria of cfDNA extraction, library construction, sequencing, and fetal DNA concentration (<4%) were removed. Pregnant women identified as high risk in NIPT results were further arranged for IPT to perform fetal karyotype analysis for trisomy 21, 18, and 13.

### IPT

Different IPT methods are selected at different gestational weeks. Chorionic villus sampling is often performed between 11 and 14 weeks of gestation. A sample of the developing placenta is collected, and the fetal chromosome karyotype is evaluated under continuous ultrasound guidance. Amniocentesis is often performed between 16 and 26 weeks of gestation, in which a needle is inserted through the pregnant woman's abdomen under ultrasound guidance to extract 20-30 mL of amniotic fluid for testing. After sampling, cell culture is performed for karyotype analysis. High-risk pregnant women with confirmed positive results from NIPT are provided with intervention guidance. In contrast, those with false positive and low-risk results are followed up to understand their pregnancy outcomes and newborn health status.

### Data collection

Collect basic information about pregnant women, including age, gestational weeks, NIPT screening results, results of IPT, and follow-up results of fetal chromosomal abnormalities; track pregnancy outcomes through phone follow-ups; and record developmental abnormalities.

### Statistical analysis

This paper's statistical data analysis was performed by applying SPSS 20.0 software. The measurement data were expressed as mean ±SD, and the two independent samples T-test were used for comparative analysis. Counting data were expressed as adoption rates and percentages, and differences between groups were compared using Chi-square tests or Fisher's exact tests. A multivariate logistic model was used to analyse and screen the independent influencing factors of inconsistent NIPT results. Taking *P*<0.05 was considered statistically significant.

For the outlier value, we use the box diagram's interquartile distance (IQR) to detect outliers, and IQR is the D-value between the first and third quantile of the variable. One and a half times the IQR was considered the standard. We stipulated that values exceeding (upper quartile add one and a half times IQR distance or lower quartile minus 1.5 times IQR distance) were outliers, and the outliers screened out were treated as missing values.

For missing value data, we treat missing values according to the percentage of missing values if the incidence is over 2%. For continuous quantitative data, the mean or median of the variable was used for missing value interpolation. In contrast, for qualitative data, the mode attribute of the variable (i.e., the value with the highest frequency) was used for missing value interpolation. If the missing ratio is greater than 2% and less than 15%, the missing value of the variable is supplemented by multiple interpolation methods. All variables had a missing percentage of less than 15 per cent.

## Results

### NIPT and IPT results

**Figure 1:**
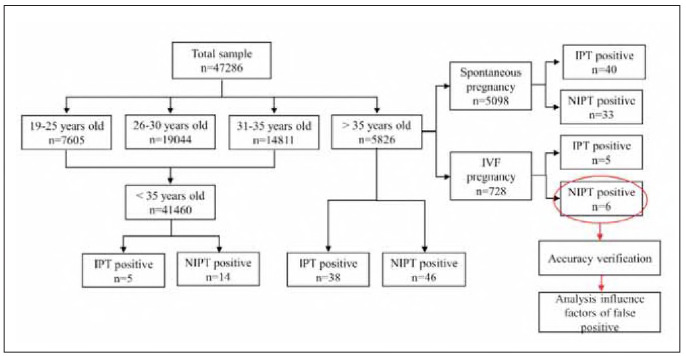
Research flow chart.

The total sample size, the number of participants in each age group, and the number of participants aged >35 who received IVF are shown in Figure 1. 47286 samples were included in this study, of which 7,605 were aged 19-25, 19,044 were aged 26-30, 14,811 were aged 31-35, and 5,826 were >35. There were 728 cases of IVF, including 5 cases of IPT-positive and 6 cases of NIPT-positive (Figure 1).

### Performance of NIPT for detecting chromosomal abnormalities

The positive rates of IPT and NIPT in different age ranges were sorted out, and the specific values were as follows: 19-25 age range (IPT 0.039%, NIPT 0.092%), 26-30 age range (IPT 0.005%, NIPT 0.026%), 31-35 age range (IPT 0.007%, NIPT 0.014%), the age >35 (IPT 0.652%, NIPT 0.789%). As pregnant women aged >35 are usually considered advanced maternal age, further analysis compared the positive detection rate between advanced and appropriate-age pregnant women. The occurrence of chromosomal abnormalities in two age groups, detected by IPT and NIPT, was analysed. The results of chromosomal abnormality occurrence indicated that the positive rates of IPT and NIPT in advanced-age pregnant women increased significantly (*P*<0.0001). However, there is no statistically significant difference in the detection rates between IPT and NIPT in advanced and appropriate-age pregnant women (*P* =0.4436 and 0.0635, respectively) (Table 1). These results suggest that NIPT has the same detection efficiency for chromosomal abnormality compared with IPT in northwest China.

**Table 1:** Comparison of the positive detection rate between advanced and appropriate-age pregnant women.

Group	IPT		NIPT		*P*
	IPT+	Total	NIPT+	Total	
Appropriate-age	5	41460	14	41460	0.0635
Advanced age	38	5826	46	5826	0.4436
*P*	<0.0001		<0.0001		

### The detection of chromosomal abnormalities between IVF and spontaneous pregnancies in pregnant women aged >35

Comparison of chromosomal abnormalities in pregnant women aged >35 with IVF and SP using IPT and NIPT detection, respectively. In the case of chromosome abnormality in pregnant women aged 35, the positive rate of women aged >35 by IPT was 0.65% spontaneous pregnancy and 0.69% IVF (*P* = 0.8071). NIPT test found that the positive rate of pregnant women aged >35 with spontaneous pregnancy was 0.78%, and that of women with IVF was 0.82% (*P*=0.8239), as shown in Table 2. These results suggest that there is no significant difference in chromosome abnormality rates between elderly pregnant women with IVF and SP Further, the results show that there is no significant difference between the positive rates detected by the NIPT and IPT in elderly pregnant women undergoing IVF and SP (*P*=0.7735 and 0.4812, respectively).

**Table 2:** Comparison of chromosomal abnormalities in pregnant women aged >35 with IVF and spontaneous pregnancies. ^1^ SP = Spontaneous pregnancy; ^2^ IVF = IVF pregnancy

Observation index		SP1 & Age>35 (n = 5098)	IVF2 & Age>35 (n=728)	*P*
IPT	Positive (case)	33	5	0.8071
	Negative (case)	5065	723	
	Detection rate (%)	0.65	0.69	
NIPT	Positive (case)	40	6	0.8239
	Negative (case)	5058	721	
	Detection rate (%)	0.78	0.82	
*P*		0.4812	0.7735	

### Screening efficacy of NIPT for three chromosome abnormalities in pregnant women aged >35 with IVF and spontaneous pregnancies

In this NIPT test, the number of high-risk cases for T21 chromosomal abnormality IVF pregnancy was 3, while for spontaneous pregnancy, it was 24. The number of high-risk cases for T18 chromosomal abnormality in IVF pregnancy was 1, while for spontaneous pregnancy, it was 9. The number of high-risk cases for T13 chromosomal abnormality in IVF pregnancy was 2, while for spontaneous pregnancy, it was 7. For pregnant women aged >35 with IVF, NIPT had a detection sensitivity, specificity, and positive predictive value of 99.72%, 99.78%, and 66.45%, respectively, for detecting common chromosomal aneuploidies (T21, T18, T13). The detection rates for T21 decreased, and T13 increased in IVF (P values of 0.044 and 0.0019, respectively). Detection rates of T18 showed no statistically significant difference compared to women who conceived naturally (P >0.05), as shown in Table 3.

**Table 3:** Efficacy of NIPT screening in two groups of pregnant women aged >35 with chromosome abnormalities. ^1^ SP = Spontaneous pregnancy; ^2^ IVF = IVF pregnancy; ^3^ CI = Confidence interval

Chromosomal <br>abnormality	T21		*P*	T18		*P*	T13		*P*	Total		*P*
	SP^1^ <br>(n=24)	IVF^2 ^<br>(n=3)		SP <br>(n = 9)	IVF <br>(n = 1)		SP<br>(n=7)	IVF <br>(n = 2)		SP <br>(n=40)	IVF <br>(n = 6)	
Positive rate (%)	0.47	0.41	0.044	0.177	0.14	0.111	0.137	0.27	0.019	0.78	2.47	0.975
Sensitivity (%)	99.74	88.50	0.490	90.45	100.56	3.002	101.08	23.98	0.568	89.74	99.72	9.289
Specificity (%)	94.78	95.44	0.100	99.33	103.45	5.624	98.65	78.66	0.178	84.86	99.78	0.223
Positive <br>predictive value <br>(95%CI^3^)	80.75	88.34	0.778	76.53	31.48	4.893	18.32	0.00	1.997	75.43	66.45	4.556

### Univariate analysis of inconsistent NIPT results for chromosome abnormalities in pregnant women aged >35 with IVF

Univariate analysis of inconsistent NIPT results for chromosome abnormalities in pregnant women aged >35 with IVF showed significant differences in mean gestational age, yield, AFP and free β-HCG between the IVF-positive group and the IVF false-positive group (*P*<0.05). However, the two groups had no significant differences in body weight, BMI and number of pregnancies (*P*<0.05), see Table 4.

**Table 4:** Univariate analysis of inconsistent NIPT results for chromosome abnormalities in pregnant women aged >35 with IVF

Observation index		Consistent cases<br>(n = 624)	inconsistent cases<br>(n = 104)	χ^2^/t	P
Body weight (kg)		61.38±9.08	62.22±7.65	0.166	0.853
BMI (kg/m^2^)		22.80±3.11	24.10±3.45	0.677	0.508
Mean gestational age		16.44±4.90	23.00±5.11	2.282	0.037
AFP (MoM)		0.99±0.40	1.50±0.34	2.555	0.021
free β-HCG (MoM)		1.28±1.09	2.48±0.70	2.686	0.016
Yield (cases)	1	312	104	10.000	0.01
	2	312	0		
Number of pregnancies<br>(cases)	1	416	104	2.700	0.086
	2	208	0		
	3	0	0		
Observation index		Consistent cases<br>(n = 624)	Consistent cases<br>(n = 624)	χ2/t	P
Body weight (kg)		61.38±9.08	62.22±7.65	0.166	0.853
BMI (kg/m^2^)		22.80±3.11	24.10±3.45	0.677	0.508
Mean gestational age		16.44±4.90	23.00±5.11	2.282	0.037
AFP (MoM)		0.99±0.40	1.50±0.34	2.555	0.021
free β-HCG (MoM)		1.28±1.09	2.48±0.70	2.686	0.016
Yield (example)	1	3	1	10.000	0.01
	2	3	0		
Number of pregnancies	1	4	1	2.700	0.086
	2	2	0		
	3	0	0		

### Logistic regression analysis of inconsistent NIPT results of chromosome abnormalities in pregnant women aged >35 with IVF

The factors with significant differences mentioned above were assigned as independent variables, and whether the diagnosis failed as dependent variables, with values of 1 and 2, respectively. The independent risk factors for NIPT results inconsistency in detecting chromosomal abnormalities in pregnant women aged >35 with chromosomal abnormalities IVF were observed. It was found that the average gestational week and AFP were independent risk factors that influenced the failure of NIPT diagnosis (*P*<0.05), as shown in Table 5.

**Table 5:** Logistic regression analysis of NIPT results inconsistency in detecting chromosomal abnormalities in pregnant women aged >35 with IVF ^1^ B = Estimate; ^2^ SE = Standard error; ^3^ Wald = Z-squared; ^4^ OR = Odds ratio

Influencing factor	B^1^	SE^2^	Wald^3^	P	OR^4^	OR(95% CI^5^)
Mean gestational age	2.877	1.198	5.771	0.0.16	17.769	1.699-185.859
yield	-2.746	1.206	5.187	0.063	0.064	0.006-0.682
AFP	-2.153	0.899	7.817	0.005	0.081	0.014-0.472
free β-HCG	-1.373	0.615	4.987	0.326	0.253	0.076-0.845

## Discussion

IVF has become increasingly mature and an important and routine animal reproductive biotechnology. There are few reports on the diagnosis of chromosomal abnormalities in IVF, especially in northwest China. It is well-known that advanced maternal age increases the risk of meiotic/embryonic or offspring aneuploidy [34]. However, the sensitivity of using maternal age alone as an indicator is very low, with a high false-positive rate [35]. Moreover, although increasing maternal age does increase the risk for T21, T13, and T18, it does not represent risk factors for other aneuploidies, such as sex chromosome aneuploidies or triploidy [36]. This study examined the detection of chromosomal abnormalities in pregnant women of different ages (19-25 years, 26-30 years, 31-35 years, and >35 years) using IPT and NIPT. It was confirmed that advanced maternal age was a sensitive factor for fetal chromosomal variations. Therefore, the results of this study match expectations. The T21, T18, and T13 detection ratios of NIPT positivity in advanced-age pregnant women is 0.79%, which is similar to the previously reported in southern China [37].

The purpose of prenatal diagnosis is to reduce the incidence and prevalence, which have a significant impact on the psychological and economic lives of patients and their parents, as well as being a financial burden on the national health system [38]. Chromosomal abnormalities have been thoroughly studied since Down syndrome was characterised as trisomy [39]. Technological innovations have made detecting minor genetic abnormalities, including many single-gene diseases, possible over the past decades [40]. Since the introduction of NIPT in 2011, more than 2 million NIPTs have been performed [41]. With the development of genetic testing, it is increasingly vital to ensure the health of offspring, whether through spontaneous pregnancy or assisted reproductive technologies, with the primary purpose being to reduce the chance of developing recessive diseases before pregnancy [42] [43] [44]. This study used NIPT detection technology to compare the favourable detection rates of chromosomal abnormalities in older and younger pregnant women. The results showed a statistically significant difference (*P*<0.05) between the two age groups.

As multiple studies have demonstrated that the incidence of aneuploidy increases with maternal age [45] [46] [47], we selected pregnant women aged >35 who underwent assisted reproductive technology to analyse the effectiveness of NIPT. It is well known that pregnant women undergoing assisted reproductive technology are more likely to detect chromosomal abnormalities in fetuses [48]. Therefore, we continued to explore the value of NIPT for screening fetal chromosomal abnormalities in older women undergoing assisted reproductive technology. Firstly, we have confirmed that NIPT can effectively detect common chromosomal abnormalities in SP and IVF pregnant women. NIPT has high sensitivity and specificity in screening for T21, T13, and T18. In the following study, we selected pregnant women with chromosomal abnormalities who underwent assisted reproductive technology or spontaneous pregnancy and detected patients with T21, T13, and T18 who underwent assisted reproductive technology or spontaneous pregnancy. The results showed significant differences in the NIPT diagnosis of T21 and T13.

Next, we explored the reasons for false-positive results in NIPT diagnosis. Studies have shown that AFP and free β-HCG are commonly used markers for screening fetal blood for chromosomal abnormalities [49] [50] [51]. In this study, we found that these two markers showed significant differences in expression among older pregnant women with chromosomal abnormalities from spontaneous or IVF pregnancy. However, when included in the multifactor analysis, only AFP was identified as a risk factor affecting the efficiency of NIPT detection. As AFP is a commonly used marker for mid-pregnancy screening [52], we speculate that factors causing inconsistent NIPT results in mid-pregnancy may be related to abnormal AFP levels. Placenta accreta allows AFP in fetal blood to enter maternal blood directly, so the level of AFP in its serum can be significantly increased, reaching 2 to 5 times that of the normal control group. Therefore, if the AFP level in pregnant women's serum is elevated after excluding fetal malformations, placental haemorrhage, and other conditions, the possibility of placental implantation should be considered. In addition, factors such as mean gestational age, weight, BMI, and the number of pregnancies did not have as much impact on detection as expected. Only mean gestational age and parity were independent factors affecting the diagnostic results of NIPT. The study suggests that achieving adequate cfDNA concentration is an essential guarantee for the success of NIPT detection [53], but this data was not statistically analysed in this study. In future studies, we will further explore this aspect.

In conclusion, NIPT can effectively detect common chromosome aneuploidy in pregnant women with IVF in northwest China. However, the mean gestational age, AFP the number of pregnancies, and other factors affecting detection accuracy must be considered.

## Dodatak

### Ethics approval and consent to participate

The hospital ethics committee of the Maternal and Child Health Hospital of Urumqi City approved the study design and any potential ethical issues. The Ethical Approval Number: XJFYLL2023049.

### Data availability statement

The datasets generated during and/or analysed during the current study are available from the corresponding author upon reasonable request.

### Funding

This study was supported by grants from the Natural Science Foundation of Xinjiang Uygur Autonomous Region (No. 2020D01A26) Science and Technology Innovation Team (Tianshan Innovation Team) project (No. 2022TSYCTD0016).

### Authors' contribution

All authors contributed to the study's conception and design. Material preparation, data collection, and analysis were performed by Shuyuan Xue, Lixia Wang, ZhenYu, Guifeng Ding, and PenggaoDai. The first draft of the manuscript was written by Shuyuan Xue, Jingying Zhu and LeFeng. All authors commented on previous versions of the manuscript. All authors read and approved the final manuscript.

### Informed consent

Informed consent was obtained from all individual participants included in the study. The authors affirm that human research participants provided informed consent for the publication of all the images and Figures. Patients and families had signed informed consent.

### Conflict of interest statement

All the authors declare that they have no conflict of interest in this work.
